# Vitamin K and childhood cancer: analysis of individual patient data from six case–control studies

**DOI:** 10.1038/sj.bjc.6600007

**Published:** 2002-01-07

**Authors:** E Roman, N T Fear, P Ansell, D Bull, G Draper, P McKinney, J Michaelis, S J Passmore, R von Kries

**Affiliations:** Leukaemia Research Fund, Institute of Epidemiology, University of Leeds, 30 Hyde Terrace, Leeds LS2 9LN, UK; Cancer Epidemiology Unit, Imperial Cancer Research Fund, Radcliffe Infirmary, Oxford OX2 6HE, UK; Childhood Cancer Research Group, 57 Woodstock Road, Oxford OX2 6HJ, UK; Information and Statistics Division, Trinity Park House, South Trinity Road, Edinburgh EH5 3SQ, UK; Institute of Medical Statistics and Documentation, Mainz, Germany; Institute for Social Paediatrics and Adolescent Medicine, Munchen, Germany

**Keywords:** childhood cancer, intramuscular administration, leukaemia, vitamin K

## Abstract

To investigate the hypothesis that neonates who receive intramuscular vitamin K are at an increased risk of developing cancer, particularly leukaemia, a pooled analysis of individual patient data from six case–control studies conducted in Great Britain and Germany has been undertaken. Subjects comprised 2431 case children diagnosed with cancer before 15 years of age and 6338 control children. The retrospective assessment of whether or not an individual baby received vitamin K is not straightforward. In many cases no record was found in stored medical notes and two types of analysis were therefore conducted; in the first it was assumed that where no written record of vitamin K was found it had not been given, and in the second, where no written record of administration was found, information on hospital policy and perinatal morbidity was used to ‘impute’ whether or not vitamin K had been given. In the first analysis, no association was found between neonatal administration of intramuscular. vitamin K and childhood cancer: odds ratios adjusted for mode of delivery, admission to special care baby unit and low birth weight were 1.09 (95% confidence interval 0.92–1.28) for leukaemia and 1.05 (0.92–1.20) for other cancers. In the second analysis, the adjusted odds ratios increased to 1.21 (1.02–1.44) for leukaemia and 1.10 (0.95–1.26) for other cancers. This shift did not occur in all studies, and when data from the hypothesis generating Bristol study were excluded, the adjusted odds ratios for leukaemia became 1.06 (0.89–1.25) in the first analysis and 1.16 (0.97–1.39) when data on prophylaxis imputed from hospital policy and perinatal morbidity were used. We conclude that whilst the broad nature of the diagnostic groups and the poor quality of some of the vitamin K data mean that small effects cannot be entirely ruled out, our analysis provides no convincing evidence that intramuscular vitamin K is associated with childhood leukaemia.

*British Journal of Cancer* (2002) **86**, 63–69. DOI: 10.1038/sj/bjc/6600007 www.bjcancer.com

© 2002 The Cancer Research Campaign

## 

The efficacy of intramuscular (I.M.) vitamin K prophylaxis became a controversial topic when Golding *et al* (1992) reported that children who received it by this route were almost three times as likely to develop leukaemia as children who received it orally or not at all. Although subsequent studies failed to confirm these findings (Ekelund *et al*, 1993; Klebanoff *et al*, 1993; Olsen *et al*, 1994; Ansell *et al*, 1996; von Kries *et al*, 1996; Roman *et al*, 1997; McKinney *et al*, 1998; Parker *et al*, 1998; Passmore *et al*, 1998a,b), inconsistencies in their results have left lingering doubts about the safety of I.M. administration (Kaufman, 1999). As a consequence, a wide variety of prophylactic policies now operate in hospitals throughout the UK (Zipursky, 1996; Tripp and McNinch, 1998; Ansell *et al*, 2001).

In order to investigate this topic in more depth, a pooled analysis of individual patient data from the six major case–control studies (Golding *et al*, 1992; Ansell *et al*, 1996; von Kries *et al*, 1996; Roman *et al*, 1997; McKinney *et al*, 1998; Parker *et al*, 1998; Passmore *et al*, 1998a) has been undertaken. The principal aims were to use consistent definitions of exposure and potential confounders to:

- determine whether or not there was an association between neonatal administration of I.M. vitamin K and childhood cancer, particularly leukaemia;
- examine the degree to which the results varied with definition of vitamin K exposure, potentially confounding factors (e.g. mode of delivery), and hospital of birth.

### Data description and methods

Details of the six studies contributing data to these analyses are summarized in Table 1

**Table 1:**
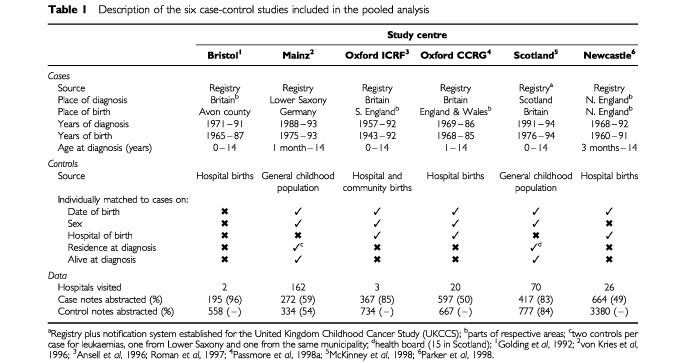
Description of the six case-control studies included in the pooled analysis

. Briefly, all studies ascertained cases from population-based cancer registries, four selecting controls from populations of hospital births (Bristol, Oxford ICRF, Oxford CCRG and Newcastle) and two from populations of children who were alive and cancer-free at the age that their corresponding case was diagnosed (Mainz and Scotland). The control group in the Bristol study comprised every 300th birth entered into the hospital delivery registers during the study period (1965 to 1987), whilst the other five groups individually matched cases to one or more controls on date of birth (month and year), and all but the Newcastle group matched on sex. The differing control sources meant that Oxford ICRF, Oxford CCRG and Newcastle individually matched controls to cases on hospital of birth, whereas Mainz and Scotland matched on region of residence at the time of diagnosis.

Studies that chose controls from hospital births visited fewer hospitals than those that chose controls from the general childhood population; the two extremes being Bristol and Mainz where the number of hospitals visited were two and 162 respectively (Table 1). The proportion of cases whose obstetric notes were found and abstracted varied from one study to another; being highest for Bristol at 96% and lowest for Newcastle at 49%. Within the two studies that selected controls from the general childhood population, the proportions of cases and controls whose obstetric notes were successfully traced were similar: 59 and 54% respectively for Mainz and 83 and 84% respectively for Scotland. Analogous figures cannot be calculated for the four studies that chose controls from populations of hospital births.

For the purposes of the pooled analysis, each research group provided anonymized individual computerized records containing information on vitamin K exposure and an agreed list of variables including: hospital of birth, date of birth, sex, gestational age, birth weight, birth order, pregnancy order, mode of delivery, apgar score and whether the baby was admitted to a special care baby unit or nursery. Prior to the pooled analyses, individual published results – using the same dataset, analytical procedures and adjusting variables as in the original study reports – were replicated and confirmed for all studies on receipt of their data (results not shown).

## THE POOLED DATASET

To standardize the data across the six studies, some subjects included in the original analyses were excluded from the pooled dataset (Table 2

**Table 2:**
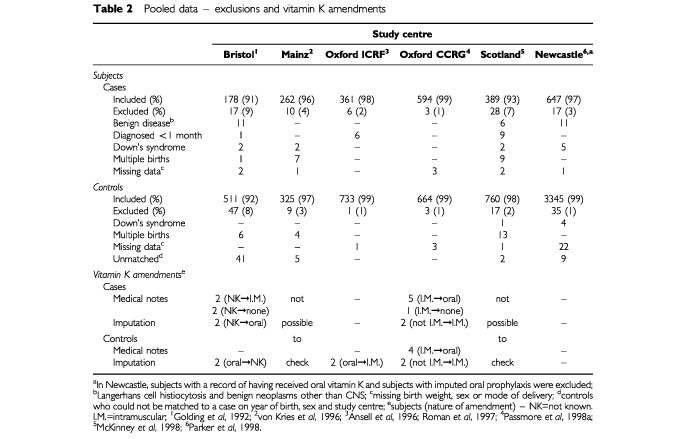
Pooled data – exclusions and vitamin K amendments

). The exclusions comprised cases diagnosed with Langerhans cell histiocytosis (21 cases); benign neoplasms (other than benign neoplasms of the central nervous system) (seven cases); or any malignancy diagnosed during the first month of life (16 cases). Subjects (cases and controls) were also excluded if they had Down's syndrome (11 cases and five controls); were part of a multiple birth (17 cases and 23 controls); had an unknown or implausible birth weight (seven cases and six controls); an unknown mode of delivery (two cases and two controls); or missing information on sex (19 controls). A further 57 controls were excluded because the study centre/sex/year-of-birth strata to which they were assigned contained no cases (see ‘The pooled analysis’). Overall, 3% of cases and 2% of controls were excluded from the pooled analysis, the final dataset comprising 2431 cases and 6338 controls.

Certain amendments were made to the vitamin K exposure variables from three study centres following examination of the computerized records (Table 2). The centres agreed all changes, which arose as errors in the original data during computation of imputed variables. Such inconsistencies could not be examined in the population-based studies.

Information about vitamin K exposure is given in Table 3

**Table 3:**
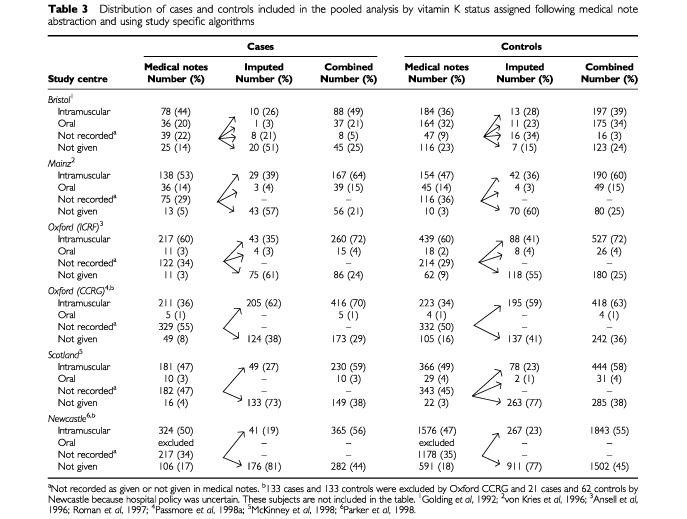
Distribution of cases and controls included in the pooled analysis by vitamin K status assigned following medical note abstraction and using study specific algorithms

: the frequencies differ from those in the published reports because of the exclusions and amendments given in Table 2. When information about vitamin K administration was not found in medical records, all groups created an imputed prophylaxis variable. For cases and controls, the central column gives the results of this imputation and the final column shows the total when medical note and imputed data are combined.

The size of the ‘not recorded’ group, and the algorithms used to impute prophylaxis (given/not given) and associated route of administration (oral/intramuscular) varied from one centre to another. Briefly:

- Bristol, Oxford CCRG and Newcastle imputed when hospital policy was reported to have been to give vitamin K to all babies by a specified route (universal) and when reported to have been to give I.M. vitamin K to ‘high risk’ neonates only (selective). In the case of the latter, factors affecting perinatal morbidity (such as mode of delivery, birth weight and admission to a special care baby unit) were reviewed and vitamin K status assigned. Neonates with a written or imputed record of having received oral vitamin K were excluded from the Newcastle study.
- Oxford ICRF and Scotland only imputed that vitamin K had been given when current hospital staff reported that the policy had, at the time the baby was born, been universal. Factors affecting perinatal morbidity were not used to impute administration when hospital policy was reported to have been selective, the advice of current hospital staff within the individual study regions being that, in such circumstances, information about vitamin K was only written down when it was, in fact, given.
- Mainz did not make a distinction between babies born at hospitals with different vitamin K policies. Instead, details about vitamin K exposure were derived from the closest child in the birth register with the same mode of delivery and perinatal morbidity as the index: it being assumed that the vitamin K status of the study child was the same as the surrogate. If this was uninformative, an opinion about the likelihood of administration was sought from medical personnel employed at the hospital at the time of the study child's birth (midwife, doctor or nurse). If this could not be achieved, medical personnel employed elsewhere were consulted.

## THE POOLED ANALYSIS

As is evident from Tables 1, 2 and 3, there are substantial differences between the individual studies – with respect to their designs, outcomes considered and exposure definitions. In the pooled analysis, in order to use as much of the data as possible, the original matching was ignored – instead all analyses took into account study centre, sex and year of birth. However, because of the large number of strata generated using logistic regression (six study centres, two sexes and 56 years of birth – 672 strata in total) the original controls provided by the centres were frequency matched to cases on year of birth, sex and study centre and relative risks estimated as odds ratios, using standard conditional logistic techniques (Breslow and Day, 1980; Selvin, 1996). This approach, which was used by the Mainz group (von Kries *et al*, 1996), produces similar results to a logistic regression model but is computationally more efficient (Neuhäuser and Becher, 1997). In all analyses, risk is assessed in relation to those known or imputed not to have received I.M. vitamin K (not given or oral); for each odds ratio, approximate 95% confidence intervals (Wald test based) and two-sided tests of statistical significance were estimated. To test for variation between centre specific odds ratios, tests for heterogeneity were calculated based on the log likelihood ratio test statistic. All data manipulation and statistical analyses were undertaken using the statistical software package STATA (Release 6.0, STATACorp, 1999).

## RESULTS

The number of exposed cases and odds ratios associated with neonatal administration of intramuscular (I.M.) vitamin K are given in Table 4

**Table 4:**
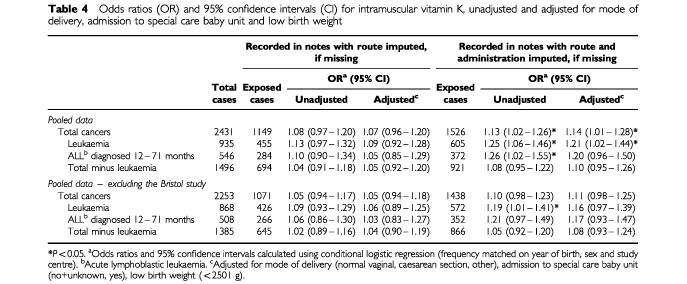
Odds ratios (OR) and 95% confidence intervals (CI) for intramuscular vitamin K, unadjusted and adjusted for mode of delivery, admission to special care baby unit and low birth weight

, those based on the total data are in the top half and those following the exclusion of data from the hypothesis generating Bristol study in the bottom half. Odds ratios on the left-hand side of the table are based on written records, assuming that where no vitamin K record was found it was not given (the route of administration, if not recorded, being based on reported hospital policy). Those on the right-hand side are based on an alternative method of imputation – those with no written record being assigned to the most likely administration category on the basis of information about perinatal morbidity and hospital prophylaxis policies. In both instances, unadjusted odds ratios and odds ratios adjusted for the potentially confounding affects of mode of delivery, admission to special care baby unit and low-birth weight are presented.

When the classification of prophylaxis (I.M. vitamin K given/not given) was based solely on the fact that written evidence of administration was available from medical notes (with route imputed if missing), the adjusted odds ratios for leukaemia and other cancers were 1.09 (95% confidence interval 0.92–1.28, *P*=0.315) and 1.05 (0.92–1.20, *P*=0.481) respectively (Table 4). When data on prophylaxis were imputed from hospital policies and perinatal morbidity using centre specific algorithms, the pooled odds ratios increased: the adjusted odds ratios for leukaemia and other cancers becoming 1.21 (1.02–1.44, *P*=0.030) and 1.10 (0.95–1.26, *P*=0.198) respectively. The odds ratios moved closer to unity when the Bristol data were excluded, with none of the adjusted odds ratios retaining their statistical significance.

The increase in the magnitude of the pooled odds ratios when administration was imputed on the grounds of hospital policy stemmed principally from two centres – Bristol and Oxford CCRG (Table 5

**Table 5:**
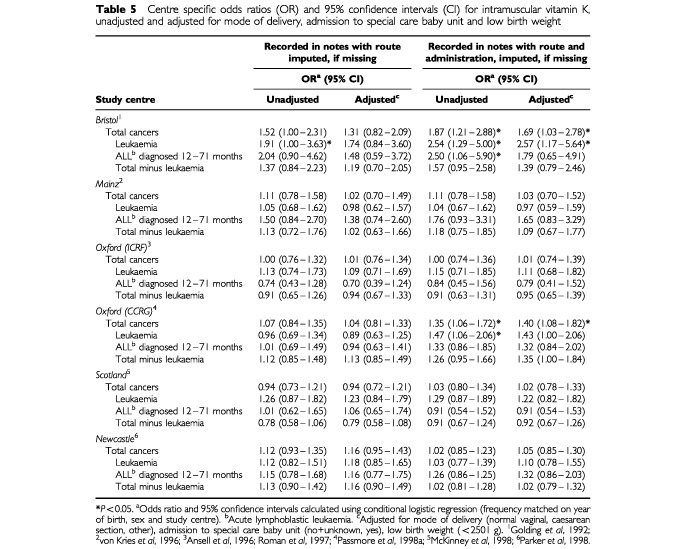
Centre specific odds ratios (OR) and 95% confidence intervals (CI) for intramuscular vitamin K, unadjusted and adjusted for mode of delivery, admission to special care baby unit and low birth weight

). The adjusted odds ratios for leukaemia increased from 1.74 (0.84–3.60, *P*=0.136) to 2.57 (1.17–5.64, *P*=0.019) in the Bristol data and from 0.89 (0.63–1.25, *P*=0.500) to 1.43 (1.00 to 2.06, *P*=0.052) in the Oxford CCRG data. In these centres, the increase, although more pronounced, was not restricted to leukaemia: the odds ratios for cancers other than leukaemia increasing from 1.19 (0.70–2.05, *P*=0.518) to 1.39 (0.79–2.46, *P*=0.257) in the Bristol data and from 1.13 (0.85–1.49, *P*=0.408) to 1.35 (1.00–1.84, *P*=0.052) in the Oxford CCRG data. Systematic changes of this type and magnitude were not evident for the four remaining centres (Table 5).

The effect of stratifying by hospital of birth is examined in Table 6

**Table 6:**
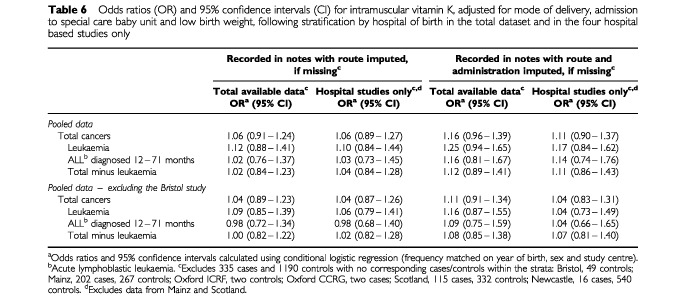
Odds ratios (OR) and 95% confidence intervals (CI) for intramuscular vitamin K, adjusted for mode of delivery, admission to special care baby unit and low birth weight, following stratification by hospital of birth in the total dataset and in the four hospital based studies only

, where odds ratios calculated using all available data and data from the four studies that selected controls from hospital births (Bristol, Oxford ICRF, Oxford CCRG and Newcastle) are presented. In general, including hospital of birth in the analysis appears to exert little effect: the adjusted odds ratios for leukaemia in the hospital-based studies (excluding the Bristol data) being 1.06 (0.79–1.41) when it was assumed that only babies with a written record of I.M. vitamin K were exposed and 1.04 (0.73–1.49) when it was assumed that babies for whom no written record was found, but for whom hospital policy/perinatal morbidity suggested that they should have received it by the I.M. route, were also classified as exposed.

## DISCUSSION

Neonatal administration of 1 mg of I.M. vitamin K is known to be effective in the prevention of both haemorrhagic disease in neonates and late onset vitamin K deficiency bleeding (McNinch and Tripp, 1991; Sutor *et al*, 1999; Zipursky, 1999). The efficacy of oral vitamin K is less certain and there have been reports that late bleeding may occur in some healthy breast-fed babies in whom no additional risk factors are identified (McNinch and Tripp, 1991; Cornelissen *et al*, 1997; Sutor *et al*, 1999; Zipursky, 1999; Wariyar *et al*, 2000). Controversy concerning prophylactic I.M. vitamin K is not new – concerns having been expressed about the proven association between Synkavit (vitamin K_2_) and kernicterus, documented cases of babies given oxytocics intended for the mother, and the use of unlicensed oral preparations. Indeed, even before the childhood cancer controversy such debate – enhanced by the growth of the natural childbirth movement – had resulted in marked variations between the policies of individual clinicians (Anonymous, 1971, 1978; Hall and Pairaudeau, 1987; Handel and Tripp, 1991; Zipursky, 1999).

The Bristol case-control study (Golding *et al*, 1992) was initiated following the unexpected associations observed in a national cohort study between childhood cancer and maternal exposure to pethidine during labour and neonatal exposure to prophylactic drugs, principally vitamin K (route unspecified) (Golding *et al*, 1990). The findings of Golding *et al* (1992), suggesting that children who received I.M. vitamin K were almost three times as likely to develop leukaemia as those given it orally or not at all, fuelled existing debate and as a consequence, throughout the UK at least, vitamin K policies were reviewed and modified, a number of epidemiological investigations initiated and laboratory-based tests and trials of alternative oral preparations and regimens undertaken.

Many of the subsequent epidemiological studies were, like the Bristol study, based on information obtained from medical records. A problem common to all was that retrospective assessment of neonatal exposure to vitamin K is not straightforward, the main reasons being:

- written records about prophylaxis can be found in several places including mothers' obstetric notes, babies' neonatal notes, delivery registers, nursing Kardex, and cot tags;
- limitations in storage space resulted in older records in some hospitals being destroyed or stored in ways that made them easily identifiable for destruction, but difficult to access for research;
- when a written record confirming administration is made, the route (oral or I.M.) is not always specified, especially when normal hospital practice is to administer vitamin K by a single route.

These difficulties led some researchers to follow the Bristol group's lead and impute information about vitamin K prophylaxis from knowledge of hospital policy. However, whilst all those investigating this topic appear to concur that route of administration could often be reliably imputed when vitamin K was recorded as given but the route was missing, a variety of approaches were adopted when no record of vitamin K administration was found in medical notes – the options ranging from assuming that when no record was found vitamin K was not given, to using clinical details such as perinatal morbidity to impute whether or not vitamin K was likely to have been given. Although the validity of using these different imputation rubrics to assess exposure has been the subject of much debate, they have never been formally investigated. Recently reported findings suggest that in the UK vitamin K policies may not be a sound basis for imputation, as hospital policies are not always followed (Ansell *et al*, 2001).

In summary, our findings suggest that neonates with a written record of having received I.M. vitamin K are no more likely to develop leukaemia, or any other cancer, before their 15th birthday than neonates without a written record of having received I.M. vitamin K. The reasons why statistically significant increases emerged when those for whom no written record was found had their exposure imputed on the basis of perinatal morbidity and hospital policy are unclear. The pooled adjusted odds ratio for leukaemia increased from 1.09 (0.92–1.28) when it was assumed that neonates for whom a written record of I.M. vitamin K was not found did not have it, to 1.21 (1.02–1.44) when the vitamin K status of neonates for whom a written record was not found was inferred from hospital policy and perinatal morbidity. This shift did not occur in all studies, and when data from the hypothesis generating Bristol study (Golding *et al*, 1992) were excluded, the adjusted odds ratios for leukaemia became 1.06 (0.89–1.25) when the analysis was based on evidence from medical notes and 1.16 (0.97–1.39) when data on prophylaxis imputed from hospital policy and perinatal morbidity were added. We conclude that the analysis of the pooled data provide no convincing evidence that I.M. vitamin K is associated with childhood leukaemia. These findings are in broad agreement with two other large investigations, whose designs differed from those included in the present pooled analysis (Ekelund *et al*, 1993; Olsen *et al*, 1994). In addition, good quality obstetric data on over 2000 children with cancer and twice as many unaffected children have been assembled as part of the United Kingdom Childhood Cancer Study (United Kingdom Childhood Cancer Study Investigators, 2000). The findings from this comprehensive population-based national study should provide additional insight.
